# Probiotic Enrichment of Jamun Juice With *Streptococcus thermophilus* : Effects on Antioxidant Activity and Quality

**DOI:** 10.1002/fsn3.70760

**Published:** 2025-08-03

**Authors:** Dorcus Nnko, Ally Mahadhy

**Affiliations:** ^1^ Department of Molecular Biology and Biotechnology, College of Natural and Applied Sciences University of Dar es Salaam Dar es Salaam Tanzania

**Keywords:** antioxidant activity, functional beverage, jamun juice, probiotics, *Streptococcus thermophilus*

## Abstract

The increasing demand for functional beverages has driven interest in incorporating probiotics into fruit juices. This study investigates the functionalization of jamun juice with *Streptococcus thermophilus* ( *S. thermophilus* ), a thermophilic probiotic bacterium, to enhance its probiotic properties and assess storage stability under varying temperature conditions. *S. thermophilus* was isolated from commercial yogurt and identified through colony morphology, Gram staining, and catalase and oxidase tests. The probiotic culture was cultivated in nutrient broth and inoculated into 250 mL of pasteurized jamun juice using 1 mL of bacterial suspension at a concentration of 1.5 × 10^8^ CFU/mL, resulting in a final concentration of 6.0 × 10^5^ CFU/mL in the juice. The functionalized juice was stored at 37°C (incubation), 25°C (room temperature), and 4°C (refrigeration) for 28 days, during which microbial load, pH, sugar content, antioxidant activity, and sensory acceptability were monitored. Results showed a significant increase in microbial load at 37°C, peaking at 10^9^ CFU/mL by Day 14. At room temperature and refrigeration, microbial growth remained minimal, with levels around 10^8^ to 10^6^ CFU/mL. The pH declined most rapidly at 37°C, and at the same temperature, sugar content decreased to 2.2 mg/mL by Day 21. Antioxidant activity, measured using the DPPH assay, was highest (82.35%) at 37°C, indicating enhanced probiotic metabolic activity. Sensory evaluations revealed improved acceptability with lower temperature storage, particularly at refrigeration temperatures, while higher temperatures led to a decrease in sensory quality. Functionalizing jamun juice with *S. thermophilus* improved its probiotic properties, with temperature playing a key role in maintaining these benefits. Refrigeration preserved both quality and probiotic stability, suggesting the potential of this probiotic jamun juice as a functional beverage. Future research could investigate its long‐term stability, sensory characteristics, and nutritional profile, including the presence of bioactive compounds.

## Graphical abstract

*S. thermophilus* isolated from commercial yogurt was successfully incorporated into pasteurized jamun juice, enhancing its probiotic and antioxidant properties. Incubation at 37°C boosted microbial growth and antioxidant activity but reduced sensory quality. Refrigeration best preserved probiotic stability and sensory acceptability, highlighting the juice's potential as a functional beverage.

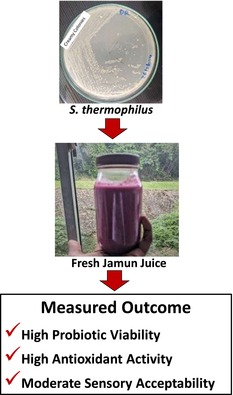

## 1 Introduction

In recent years, probiotics have gained significant attention as the importance of gut health and its impact on overall well‐being have become more widely recognized. This growing awareness has driven consumer demand for functional foods and beverages, driving substantial research into the development of probiotic‐enriched products (Koirala and Anal 2021).

Probiotics are live microorganisms that, when administered in adequate amounts, confer health benefits to the host (Aureli et al. 2011). Among the most commonly used probiotic bacteria are lactic acid bacteria (LAB), particularly those belonging to the genera *Lactobacillus* and *Streptococcus*. One of the most promising species within this group is *Streptococcus thermophilus (S. thermophilus)*, which is widely recognized for its ability to improve gastrointestinal health, enhance lactose digestion, and boost immune function (Dargahi et al. 2018). Moreover, a growing body of evidence suggests that this probiotic strain may offer a spectrum of benefits beyond digestive health, with studies indicating its potential in alleviating gastrointestinal disorders, supporting immune function, and even contributing to metabolic well‐being (Aureli et al. 2011).

*S. thermophilus* is a fermentative facultative anaerobe known for its remarkable ability to withstand harsh conditions, such as high temperatures and acidity, making it well‐suited for various food processing and manufacturing environments (Gobbetti and Calasso 2014). This resilience sets it apart from many traditional probiotic strains, which are often more delicate and sensitive to environmental stressors (Harnett et al. 2011). Consequently, *S. thermophilus* has demonstrated exceptional stability during the shelf life of various food products (Ozturk et al. 2021), which is crucial for ensuring consistent and reliable health benefits for consumers. While most studies have focused on the use of this strain in fermented dairy products, the growing trend of non‐dairy foods and beverages presents an attractive alternative for consumers with lactose intolerance or dairy allergies. For instance, the incorporation of *S. thermophilus* in carrot and beetroot juices has demonstrated successful fermentation and extended shelf life while maintaining the sensory qualities of the juices (Yoon et al. 2005). However, there is limited or no research on the development of probiotic jamun juice using *S. thermophilus* .

Jamun ( *Syzygium cumini* ), a tropical fruit native to the Indian subcontinent and East Africa (Kumar et al. 2023), has garnered attention for its rich phytochemical profile, including anthocyanins, flavonoids, and other antioxidants. These bioactive compounds contribute to its strong antioxidant capacity and potential health benefits, such as antidiabetic, anti‐inflammatory, and cardiovascular protective effects. Despite its nutritional and therapeutic potential, jamun remains underutilized in the formulation of functional beverages, particularly probiotic juices.

This study reports the development and characterization of probiotic jamun juice enriched with *S. thermophilus* isolated from commercial ASAS brand yogurt. The physicochemical properties, antioxidant activity, microbial viability, and shelf life of the developed beverage were evaluated. This resulting probiotic juice has the potential to significantly contribute to the growing market for functional beverages. Furthermore, it offers an opportunity to increase the market value of jamun fruit and encourage the use of locally fermented milk cultures.

## 2 Materials and Methods

### 2.1 Materials

Jamun fruits (5 kg), pasteurized fresh milk (1 L), and commercial ASAS brand yogurt (1 L) were purchased from local markets in Dar es Salaam, with the yogurt serving as the source of the *S. thermophilus* isolate. The materials were then transported to the Department of Molecular Biology and Biotechnology (DMBB) at the University of Dar es Salaam (UDSM), Tanzania, and stored at 4°C until further use. Nutrient agar, broth, and other reagents used in this study were all of analytical grade (Sigma‐Aldrich, St. Louis, MI, USA).

### 2.2 Methods

#### 2.2.1 Isolation of *S. thermophilus*

Two percent of yogurt (10 mL) was added to 500 mL of pasteurized fresh milk, followed by fermentation for approximately 2 days (Gobbetti et al. 2002). The fermented milk was then serially diluted 10‐fold in normal saline to obtain a 10^−3^ dilution. About 0.1 mL of each dilution was transferred in triplicate into empty Petri dishes, followed by the addition of 20 mL of warm nutrient agar (approximately 50°C) to each plate. The agar plates were allowed to solidify at room temperature and were then incubated at 37°C for 24 h (Sanders 2012). The isolates were then subcultured by streaking onto the same medium under the same conditions to obtain pure colonies.

### 2.3 Characterization of *S. thermophilus* Isolate

#### 2.3.1 Morphological and Biochemical Characterization

All morphological and biochemical tests were performed following standard protocols obtained from the Department of Molecular Biology and Biotechnology (DMBB).

For Gram staining, a loopful of pure colonies obtained from the plates was subjected to Gram staining as described by (Chamberlain and Cox 1997). The stained samples were then observed under a light microscope. Pure colonies displaying Gram‐positive, spherical coccus morphology in chains were selected as *S. thermophilus* (Sizar et al. 2023) for further biochemical characterization.

A loopful of the Gram‐positive, spherical coccus morphology in chains pure colonies was placed on a light microscope slide and tested for catalase activity according to (ASM 2011). The same isolate was subsequently tested for oxidase activity following the method described by (ASM 2019).

### 2.4 Culturing of Probiotic of *S. thermophilus*

*S. thermophilus* was cultured in nutrient broth medium as previously described by (Salmazo et al. 2023). A loopful of pure *S. thermophilus* colonies was inoculated into a conical flask containing 100 mL of sterile nutrient broth. The flask was sealed and placed on an incubator shaker at 37°C. The optical density (OD) at 600 nm was monitored every 3 h until it reached approximately OD_600_ = 1.0.

### 2.5 Preparation of Jamun Juice

About 2 kg of jamun fruits were thoroughly washed and then boiled in 1.5 L of water for 10 min, after which they were allowed to cool. The cooled fruits were deseeded and blended with 500 mL of distilled water for approximately 10 min. The resulting homogeneous mixture was then filtered into six sterile bottles, each containing about 250 mL. The bottles were stored in the refrigerator until further use (Ghosh et al. 2017).

### 2.6 Functionalization of Jamun Juice With Probiotic *S. thermophilus*

Pre‐pelleted *S. thermophilus* , recovered from the broth by centrifugation at 12,000× *g* for 5 min, was resuspended in 9 mL of sterile normal saline and adjusted to a 0.5% McFarland standard. Aseptically, 1 mL of the bacterial suspension was transferred into a 250 mL bottle containing jamun juice. This procedure was repeated for two additional bottles. Three other bottles were left uninoculated as negative controls. Each pair of inoculated and control samples was incubated at three different temperatures: 37°C in an oven, 4°C in a refrigerator, and approximately 25°C at room temperature for 28 days (Mao et al. 2023).

### 2.7 Periodic Analysis of Jamun Juice During Storage From Day 0 to Day 28

All stored juice samples were analyzed periodically at intervals of 0, 1, 7, 14, 21, and 28 days for microbial load, pH, total sugar content, antioxidant activity, and organoleptic evaluation.

For microbial load, 1 mL of stored jamun juice from each sample was serially diluted in 10‐fold steps up to a 10^−4^ dilution. The dilutions were plated in triplicate on nutrient agar and incubated at 37°C for 24 h. Colony counts were performed after incubation and expressed as CFU/mL. Independent t‐tests (*n* = 3 per group) were conducted to compare microbial load between treated and control samples at each temperature (37°C, 25°C, and 4°C). A *p* < 0.05 was considered statistically significant.

The pH of each stored sample was measured using a pH meter (HI 2211 pH/ORP Meter, HANNA Instruments, Romania). For this, 10 mL of juice was transferred from each bottle into a 25 mL beaker, and the pH was measured. Independent *t*‐tests (*n* = 3 per group) were conducted to compare pH values between probiotic and control samples at each temperature and time point. A *p* < 0.05 was considered statistically significant.

The antioxidant activity of jamun juice samples was assessed using the DPPH (2,2‐diphenyl‐1‐picrylhydrazyl) radical scavenging assay. A 0.002% DPPH stock solution was prepared in methanol, and 2 mL of this solution was mixed with 2 mL of the stored jamun juice sample. The reaction mixture was incubated in the dark at room temperature for 30 min to allow for the scavenging reaction. After incubation, the absorbance was measured at 517 nm using aUV‐Visible spectrophotometer. A blank solution containing 0.002% DPPH in methanol was used to correct for background absorbance.

The percentage of DPPH radical scavenging activity was calculated using Equation (1), as described by Baliyan et al. (2022):

(1) $$\%\text{of Scavenging activity}=\frac{A-B}{A}\times 100$$

where *A* is the absorbance of the control (DPPH solution without jamun juice), and *B* is the absorbance of the test sample (DPPH solution mixed with jamun juice). Independent *t*‐tests (*n* = 3 per group) were conducted to compare antioxidant activity between treated and control samples at each temperature on Day 21. A *p* < 0.05 was considered statistically significant.

For the determination of total sugar content in the stored probiotic juice, the phenol–sulfuric acid method was used as previously described (Dubois et al. 1956). A known volume of the juice sample (1 mL) was mixed with 1 mL of 5% phenol and 5 mL of concentrated sulfuric acid, allowed to develop color for 20 min, and the absorbance was measured at 490 nm. The absorbance values were referenced against a previously established glucose calibration curve to determine the total sugar content, as shown in Equation (2).

(2) $$\text{Absorbance}\;\left(A\right)=m\times \left[\text{sugar}\right]+b$$

where *A* is the observed absorbance of the sample, *m* is the slope, and *b* is the *y*‐intercept of the standard curve. Independent *t*‐tests (*n* = 3 per group) were performed to compare sugar concentrations between probiotic and control samples at each storage temperature on Day 21. A *p* < 0.05 was considered statistically significant.

The organoleptic evaluation of the jamun juice samples was conducted with a group of 15 untrained undergraduate students aged 20–25 years. A random single‐blind method was followed (Meilgaard et al. 2007). Panelists were provided with both control (uninoculated) and treated (inoculated with *S. thermophilus* ) juice samples. After tasting, each panelist was asked to rate the samples as either “acceptable” (taste is okay) or “not acceptable” (unpleasant).

The ratings provided by the panelists were analyzed for acceptability. The percentage of acceptable ratings for each sample was calculated by the following formula (Equation 3):

(3) $$\%\text{of Acceptable Ratings}=\frac{\text{Number of Acceptable Ratings}\;}{\text{Total Number of Ratings}\;}\times 100$$

where the Number of Acceptable Ratings is the count of panelists who found the sample acceptable, and the Total Number of Ratings is the total number of panelists who evaluated the sample.

The standard deviation (SD) for the acceptability ratings was calculated using the formula (Equation 4) for standard deviation in a binomial distribution (Illowsky and Dean 2023) given the binomial nature of the data, that is, acceptable or not acceptable.

(4) $$\mathrm{SD}=\sqrt{\frac{p\left(1-p\right)}{n}}$$

Where *p* is the proportion of successes (the percentage of acceptable ratings), and *n* is the number of panelists. Fisher's Exact Test (*n* = 15) was performed to compare sensory acceptability across sample types and storage temperatures. A *p* < 0.05 was considered statistically significant in all analyses. This test was selected due to the categorical nature of the outcome (acceptable/not acceptable) and the small sample size, which may result in low expected cell counts.

## 3 Results and Discussion

### 3.1 Isolation and Characterization of *S. thermophilus*

*S. thermophilus* was isolated from commercial yogurt and morphologically and biochemically characterized for identification. The results are presented in Table 1.

**TABLE 1:** Morphological and biochemical characteristics of the isolated *S. thermophilus* from commercial yogurt (ASAS brand).

Characteristic	Description of observations	Figures
Colony morphology	Small, circular, creamy, smooth, slightly raised compact and non‐mucoid colonies	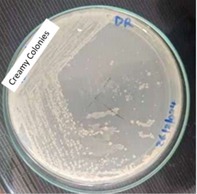
Gram staining	Gram‐positive: isolates appear blue‐purple, spherical cocci, typically arranged in chains under a light microscope	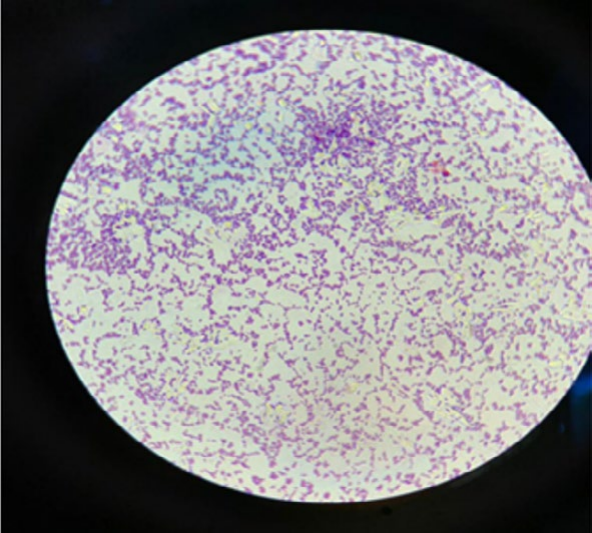
Catalase activity	Negative: no bubbles observed after adding hydrogen peroxide	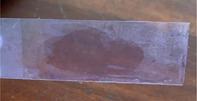
Oxidase activity	Negative: no color change when the oxidase reagent was applied	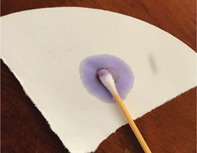

The microbiological composition of ASAS yogurt, like many commercial yogurts, primarily consists of two lactic acid bacteria species, *S. thermophilus* and *Lactobacillus delbrueckii* subsp. *Bulgaricus (L. d. bulgaricus)*. These two bacteria are essential for yogurt fermentation, working synergistically to produce lactic acid, which gives yogurt its characteristic tangy flavor and texture. *S. thermophilus* , a thermophilic bacterium, is usually present in greater numbers during the initial stages of fermentation, while *L. d. bulgaricus*, another thermophile, works in tandem with *S. thermophilus* to complete the fermentation process.

**FIGURE 1:**
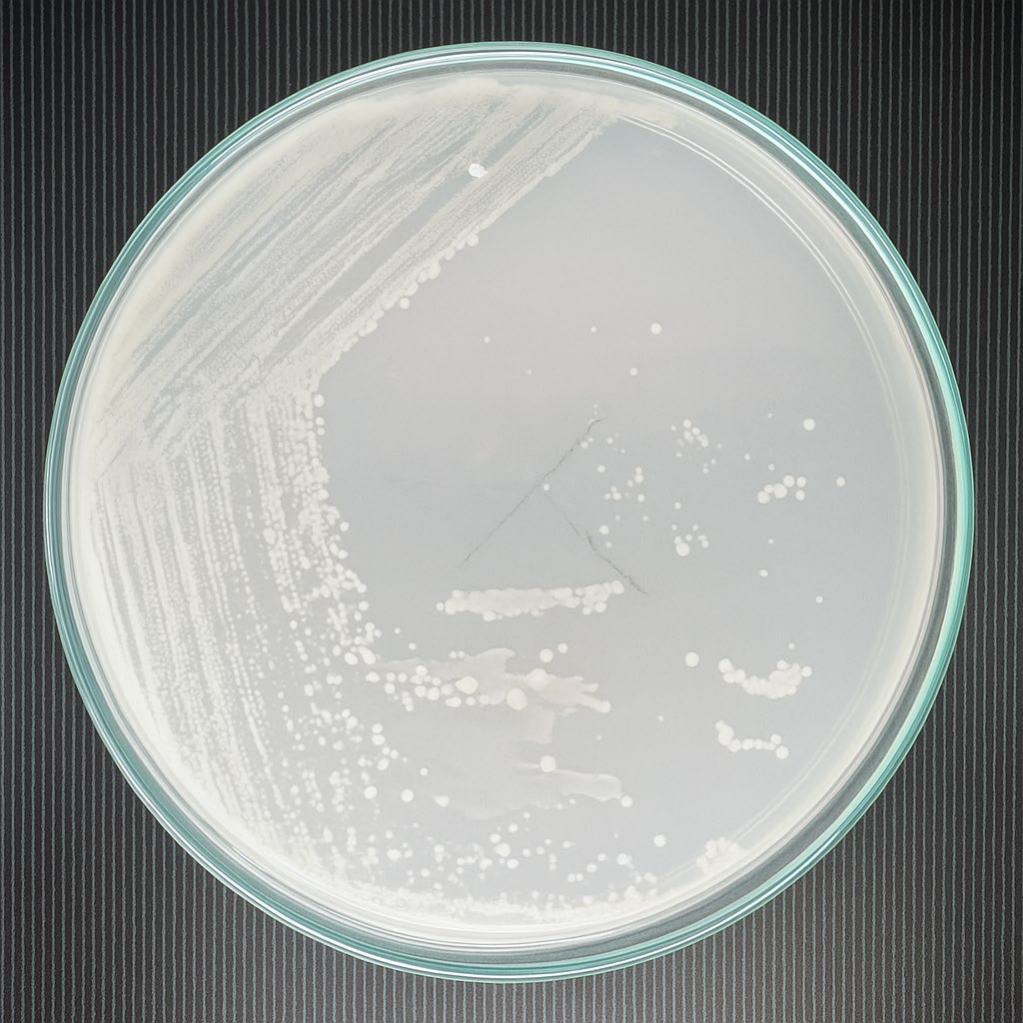
Colony morphology of predominant isolates on a nutrient agar plate.

**FIGURE 2:**
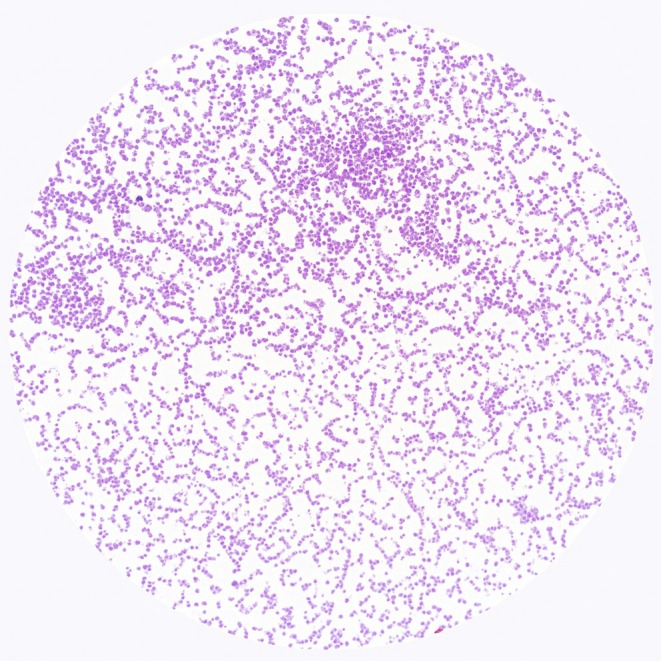
Appearance of gram‐stained presumptive *S. thermophilus* isolates under light microscopic observation (×100).

**FIGURE 3:**
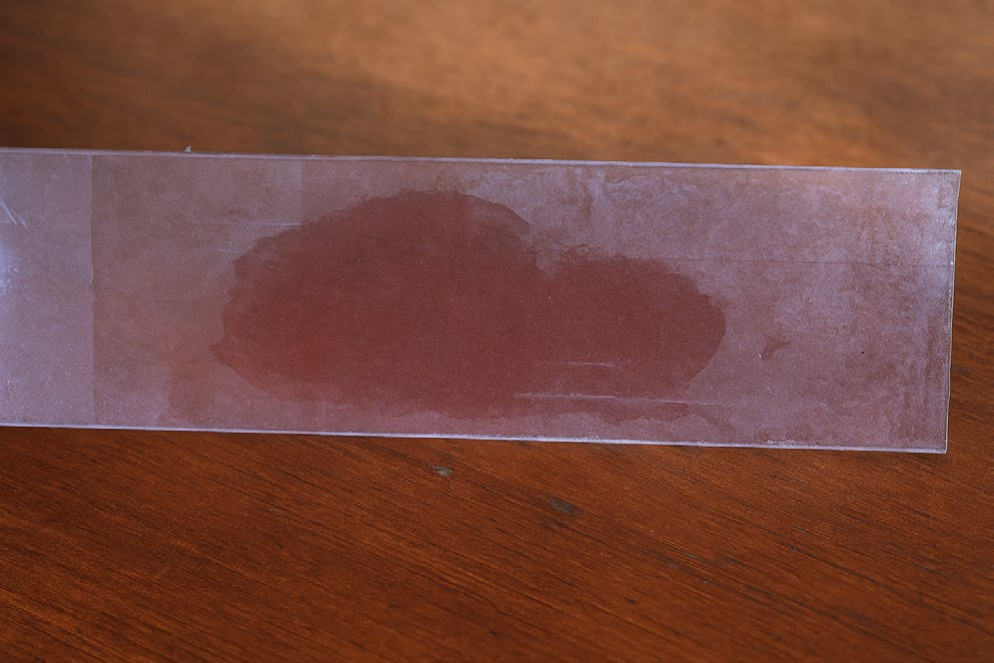
Catalase test for selected *S. thermophilus* isolates.

**FIGURE 4:**
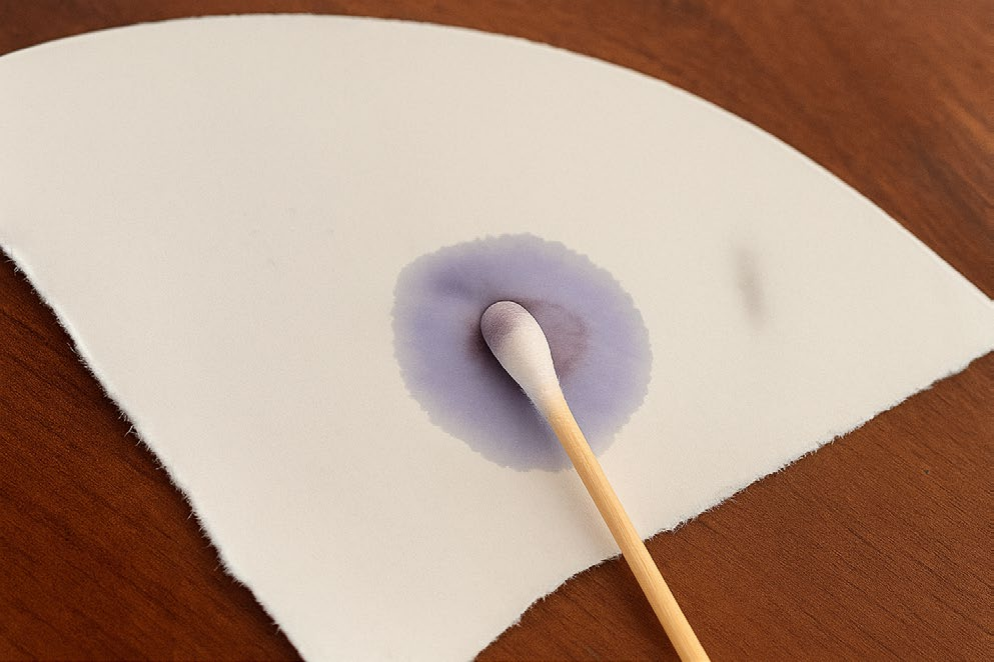
Oxidase test for selected *S. thermophilus* isolates.

In this study, *S. thermophilus* and *L. d. bulgaricus* were isolated from commercial ASAS yogurt, and their morphological and biochemical characteristics were identified. The results presented in Table 1 show that the predominant isolate on nutrient agar was *S. thermophilus* , identified through colony morphology (Figure 1), gram staining (Figure 2), and catalase (Figure 3) and oxidase tests (Figure 4). These features are typical of *S. thermophilus* , as previously documented (Gobbetti and Calasso 2014). Although *S. thermophilus* shares similar Gram‐staining reactions, as well as catalase and oxidase activities, with *L. d*. *bulgaricus*, its colony morphology is distinctly different. *S. thermophilus* typically forms small, creamy, smooth, and compact colonies, whereas *L. d. bulgaricus* produces larger, circular but slightly irregular colonies that are white or off‐white in color, with a rough, dry, or slightly mucoid texture (Yang et al. 2023).

Nutrient Agar is a general‐purpose medium that provides adequate nutrients for *S. thermophilus* , but it is less favorable for *L. d. bulgaricus* growth (Tamime and Robinson 2007). The latter bacterium is more fastidious and typically requires more specialized environments, such as those with slightly lower pH or other selective conditions (Dedysh 2023). Therefore, it is not surprising that *S. thermophilus* became the predominant isolate under the given conditions, which are favorable for its growth and not as optimal for *L. d. bulgaricus*.

Similar findings have been reported in other studies, where *S. thermophilus* was found to dominate in yogurt cultures on general‐purpose media like nutrient agar. For example, in a study by Moon and Reinbold (1976), *S. thermophilus* was consistently observed to predominate on media like nutrient agar or MRS agar, while *L. d. bulgaricus* was often less abundant or more difficult to isolate under these conditions. This supports the idea that *S. thermophilus* has a robust growth capability in a variety of media, particularly under conditions that do not favor other bacterial species like *L. d. bulgaricus*.

While classical morphological and biochemical tests provided sufficient preliminary identification of *S. thermophilus* in this study, we acknowledge the absence of molecular confirmation methods such as 16S rRNA gene sequencing. Due to resource limitations, these were not performed, and we recommend their inclusion in future studies to enhance taxonomic accuracy and verification.

### 3.2 Culturing of Probiotic of *S. thermophilus*

The desired probiotic *S. thermophilus* bacteria were mass‐cultivated in nutrient broth medium at 37°C on an incubator shaker. Growth was monitored by measuring turbidity/optical density at 600 nm using a spectrophotometer. The culture was harvested when the OD600 reached 1.0, which corresponds to the late exponential phase. This phase is ideal for harvesting active and viable cells for probiotic applications and generally reflects 10^8^–10^9^ CFU/mL (Salmazo et al. 2023).

### 3.3 Prepared Fresh Jamun Juice

**FIGURE 5:**
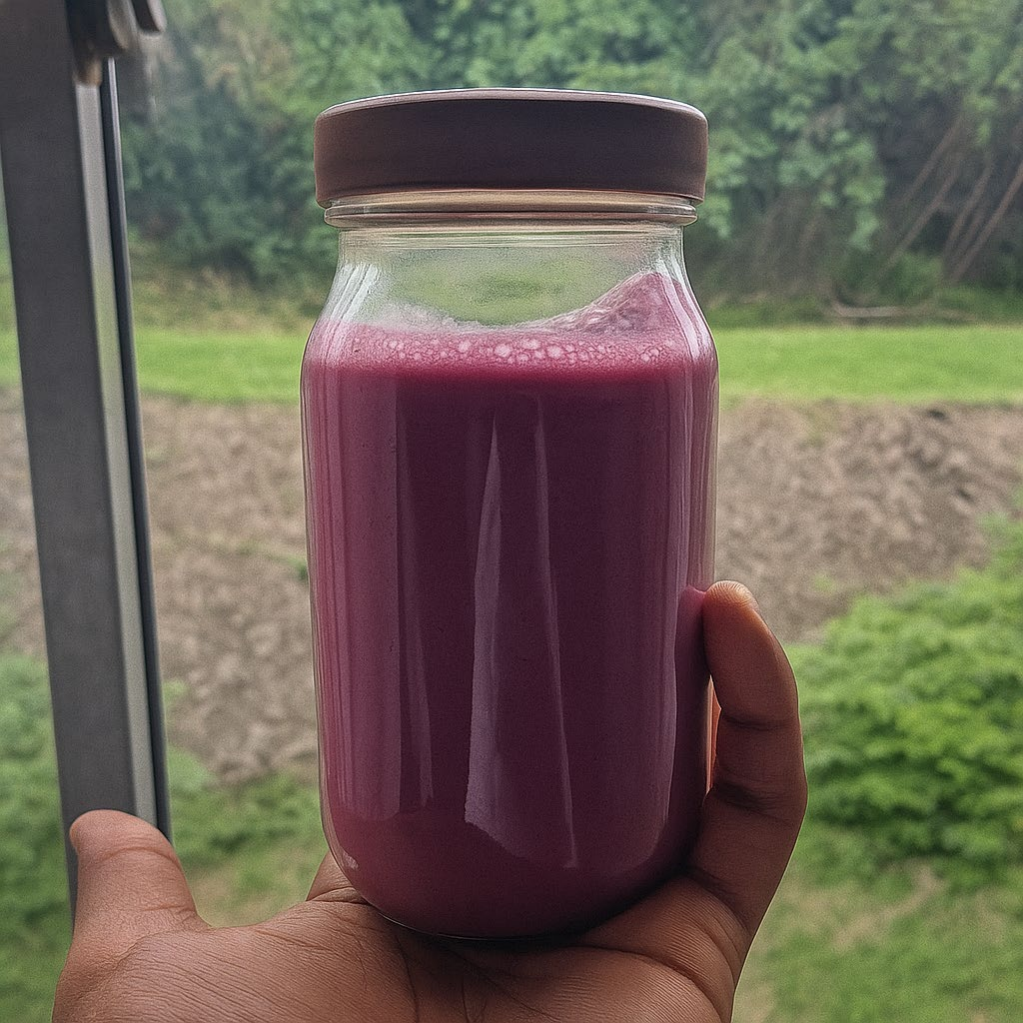
Fresh pasteurized jamun juice packed in a 250 mL bottle.

Fresh jamun fruit was blended with sterile water to obtain the juice. The juice had a sour taste, typical of fresh jamun fruits, with a pH of 4.2. This is consistent with previous studies (Ghosh et al. 2017), which have reported jamun juice with a pH ranging from 3.5 to 4.5. The juice was then thermally pasteurized by boiling for 10 min, with no additives like sugar or preservatives. The juice was packed into 25 mL bottles (Figure 5), ready for functionalization with probiotic *S. thermophilus* .

### 3.4 Functionalized Probiotic Jamun Juice With *S. thermophilus*

**FIGURE 6:**
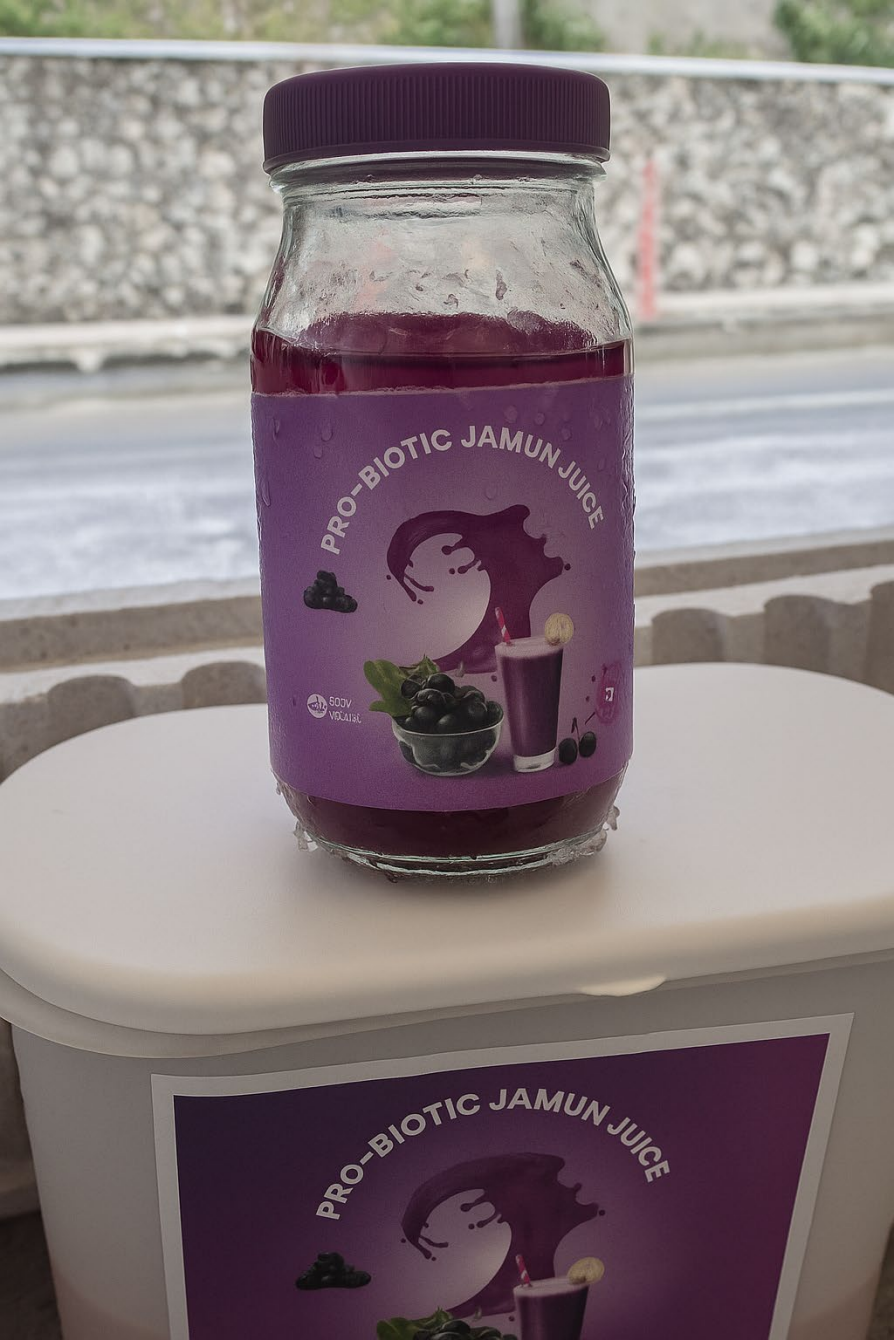
A 250 mL bottle containing functionalized probiotic jamun juice.

Approximately 1.5 × 10^8^ CFU/mL of viable *S. thermophilus* cells, recovered from nutrient broth, were introduced into each of three 250 mL bottles of pasteurized jamun juice, resulting in a final concentration of 6.0 × 10^5^ CFU/mL. The bottles were stored under different temperature conditions and compared with their uninoculated counterparts. Figure 6 shows functionalized probiotic jamun juice.

### 3.5 Characterization of Functionalized Probiotic Jamun Juice

#### 3.5.1 Microbial Load Analysis

**FIGURE 7:**
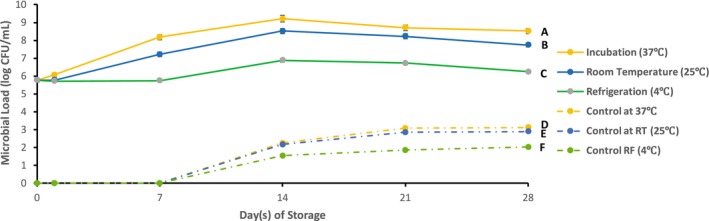
Microbial load in functionalized probiotic jamun juice stored at different temperature conditions: (A) incubator at 37°C (yellow solid line), (B) room temperature (blue solid line), and (C) refrigeration (green solid line), along with (D–F) respective control samples (dash dot lines).

The functionalized probiotic jamun juice samples, along with their respective control samples, were stored under different temperature conditions and subsequently characterized for microbial load, pH, sugar content, antioxidant activity, and sensory acceptability through organoleptic testing. The microbial load in the functionalized probiotic jamun juice samples, stored under various temperature conditions, along with their control samples, is shown in Figure 7.

After 1 day of storage, the juice stored at 37°C exhibited an increase in microbial load from the initial inoculated concentration of about 10^5^ CFU/mL to about 10^6^ CFU/mL. In contrast, no significant microbial growth was observed in the samples stored at room temperature (25°C) and under refrigeration (4°C), with the microbial counts remaining at the inoculated level of 10^5^ CFU/mL. This early increase in microbial population at elevated temperature indicates the rapid activation and growth of the inoculated *Streptococcus thermophilus* , while growth was suppressed in cooler storage conditions. This result aligns with similar studies, such as that by Béal et al. (1989), who observed a faster growth rate of probiotics in warmer conditions, while lower temperatures significantly inhibited microbial growth.

No detectable microbial growth was observed in any of the un‐inoculated control samples at the initial time point, confirming that the observed microbial populations in the probiotic samples were due to the inoculated strain. Over the following days, the microbial load continued to increase in all probiotic juice samples. Between days 7 and 14, a significant rise was recorded, reaching a plateau of approximately 10^9^ CFU/mL in the incubated (37°C) sample, 10^8^ CFU/mL in the sample stored at room temperature (25°C), and 10^6^ CFU/mL in the refrigerated (4°C) sample. These findings reflect the influence of storage temperature on microbial proliferation, with higher temperatures facilitating faster and greater growth of the probiotic strain. This is consistent with Yang et al. (2023), who demonstrated that higher incubation temperatures promote more robust growth of *Lactobacillus* strains in fermented products.

From Day 14 to Day 28, a decline in microbial load was observed in the samples stored at 37°C and room temperature, decreasing to around 10^8^ CFU/mL and 10^7^ CFU/mL, respectively. This reduction could be attributed to the depletion of nutrients or the accumulation of inhibitory metabolic by‐products over time. In contrast, the refrigerated sample exhibited an insignificant decrease, maintaining a microbial load of approximately 10^6^ CFU/mL, suggesting that low‐temperature storage helped preserve the viability and stability of the probiotic population over a longer period. Similar observations have been reported by Amstrong et al. (2016) who found that refrigeration extended the shelf life of probiotic beverages, effectively maintaining microbial stability compared to samples stored at room temperature or higher.

Meanwhile, the control samples stored under the same temperature conditions also demonstrated slight microbial growth over the 28‐day period. However, microbial load was significantly higher in treated samples compared to control samples at all temperature conditions: 37°C (*p* < 0.001), 25°C (*p* < 0.001), and 4°C (*p* < 0.001). At Day 14, colony counts were approximately 180 CFU/mL at 37°C, 140 CFU/mL at room temperature, and 30 CFU/mL under refrigeration. By Day 28, these values increased modestly to 1300 CFU/mL, 790 CFU/mL, and 100 CFU/mL, respectively. Although these values remain significantly lower than those observed in the probiotic samples, their presence indicates the survival and slow growth of heat‐resistant fungal spores or other microbes that withstood pasteurization. This phenomenon aligns with studies such as that of Shearer et al. (2002), who reported that pasteurized fruit juices often harbor heat‐resistant microorganisms, including fungi and spores, despite undergoing sterilization. Notably, microbial proliferation in the refrigerated control sample remained minimal, further supporting the effectiveness of refrigeration in inhibiting spoilage, as shown by Sameli et al. (2021) in their examination of microbial stability in refrigerated fruit juices.

### 3.6 Monitoring of pH Values of Probiotic Jamun Juice During Storage

The pH values of probiotic jamun juice samples were monitored over the storage period under three different temperature conditions: incubation (37°C), room temperature (25°C), and refrigeration (4°C). The results are shown in Table 2.

**TABLE 2:** Measured pH values of probiotic jamun juice samples stored under different temperature conditions and their respective control samples.

Storage duration (days)	Incubation (37°C)		Room temperature (25°C)		Refrigeration (4°C)	
	Probiotic	Control	Probiotic	Control	Probiotic	Control
0	4.0 ± 0.10	4.2 ± 0.08	4.0 ± 0.09	4.2 ± 0.01	4.0 ± 0.07	4.2 ± 0.03
1	3.4 ± 0.14	4.0 ± 0.13	3.6 ± 0.05	4.2 ± 0.14	4.0 ± 0.10	4.2 ± 0.07
7	3.3 ± 0.12	3.8 ± 0.09	3.5 ± 0.03	4.0 ± 0.06	3.9 ± 0.01	4.1 ± 0.05
14	3.0 ± 0.08	3.5 ± 0.08	3.2 ± 0.10	3.7 ± 0.09	3.7 ± 0.04	4.0 ± 0.10
21	2.8 ± 0.13	3.1 ± 0.17	3.0 ± 0.0	3.3 ± 0.21	3.4 ± 0.09	3.8 ± 0.08
28	2.9 ± 0.19	3.0 ± 0.11	3.0 ± 0.06	3.2 ± 0.05	3.4 ± 0.10	3.9 ± 0.01

The results indicate a general trend of decreasing pH over time in all probiotic samples, suggesting active fermentation and acid production by probiotic microorganisms (Mani‐López et al. 2014). Samples stored at higher temperatures, particularly at 37°C, exhibited a more rapid decline in pH compared to those kept at room temperature (25°C) or under refrigeration (4°C). In contrast, control samples without probiotic inoculation maintained relatively stable pH values across all storage conditions, confirming that the acidification observed in probiotic samples was due to microbial activity.

Independent t‐tests (*n* = 3 per group) revealed statistically significant differences (*p* < 0.05) in pH between probiotic and control samples at most time points, especially between Days 1 and 14 under 37°C and 25°C. For instance, at 37°C, pH values were significantly lower in probiotic samples than in controls from Day 1 (*p* = 0.0299) to Day 14 (*p* = 0.0012), with a similar trend at 25°C. However, fewer significant differences were observed at Days 21 and 28, possibly indicating a slowdown in microbial activity or stabilization of acidity.

These results suggest that probiotic jamun juice exhibits a temperature‐dependent reduction in pH over storage time, reflecting microbial viability and metabolic activity. Storage at elevated temperatures accelerates acid production, while refrigeration slows this process. These findings have important implications for product formulation, shelf life, and recommended storage conditions for maintaining probiotic functionality.

### 3.7 Evaluation of Total Sugar Content in the Stored Probiotic Jamun Juice Samples

**FIGURE 8:**
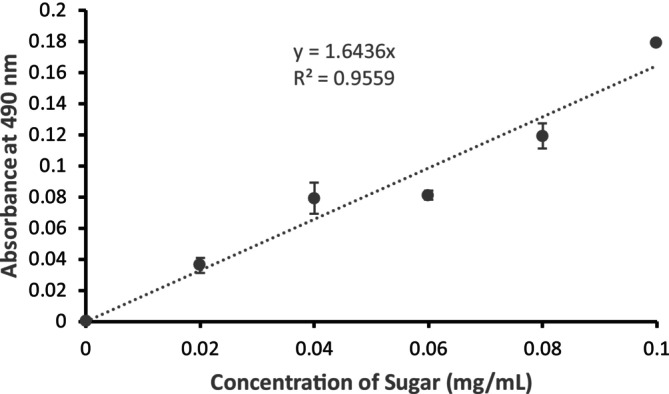
Standard calibration curve for total sugar determination using the phenol–sulfuric acid method. A series of glucose standards (0–0.10 mg/mL) were reacted with 5% phenol and concentrated sulfuric acid, and absorbance was measured at 490 nm. The absorbance values showed a linear relationship with glucose concentration (*R* ^2^ = 0.956).

The total sugar content in probiotic juice samples was determined at the beginning (Day 0) and after storage (Day 21) using the phenol‐sulfuric acid method. Day 21 was selected for post‐storage analysis as it corresponds to the phase where microbial growth had begun to decline and stabilize, indicating the likely completion of active fermentation and maximum sugar utilization by the probiotic culture. This time point provides a representative measure of the residual sugar content in the final product, which is critical for assessing its nutritional composition and shelf‐life stability. Absorbance was measured at 490 nm and quantified against a standard calibration curve (Figure 8). The corresponding sugar concentrations are presented in Table 3.

**TABLE 3:** Sugar concentration (mg/mL) in probiotic and control samples at different storage temperatures over time.

Storage duration (days)	Sugar concentration at 37°C (incubation) (mg/mL)		Sugar concentration at 25°C (room temperature) (mg/mL)		Sugar concentration at 4°C (refrigeration) (mg/mL)	
	Probiotic	Control	Probiotic	Control	Probiotic	Control
0	10.3 ± 0.01	10.2 ± 0.1	10.3 ± 0.03	10.2 ± 0.2	10.3 ± 0.01	10.2 ± 0.05
21	2.2 ± 0.18	7.4 ± 0.22	3.3 ± 0.15	8.2 ± 0.09	5.3 ± 0.20	9.6 ± 0.19

From Table 3, a clear decline in total sugar content was observed in probiotic jamun juice samples over the 21‐day storage period, with the extent of reduction varying according to storage temperature. The most pronounced decrease occurred in samples incubated at 37°C, where sugar levels dropped from 10.2 mg/mL at Day 0 to 2.2 mg/mL by Day 21, indicating active metabolic utilization of sugars by probiotic microorganisms. Samples stored at room temperature (25°C) and under refrigeration (4°C) exhibited more moderate reductions, reaching 3.3 mg/mL and 5.3 mg/mL, respectively. Control samples, lacking probiotic inoculation, retained significantly higher sugar concentrations under all storage conditions, particularly under refrigeration (9.6 mg/mL), confirming that the observed sugar depletion was primarily due to microbial fermentation.

Independent *t*‐tests (*n* = 3 per group) confirmed that sugar concentrations in probiotic samples were significantly lower than in controls at Day 21 across all temperatures (*p* < 0.001). This highlights enhanced sugar consumption by probiotics, especially at elevated temperatures. The greater reduction at 37°C aligns with increased probiotic metabolic activity, while the slower decline at lower temperatures reflects diminished fermentation rates.

These findings demonstrate a temperature‐dependent fermentation dynamic, with higher temperatures promoting probiotic activity and sugar utilization. Similar trends have been reported in fruit‐based probiotic matrices; for example, Khalid et al. (2018) observed significant sugar declines in probiotic pomegranate juice incubated at 37°C, attributed to Lactobacillus fermentation. Likewise, Østlie et al. (2005) showed greater probiotic metabolic activity and sugar consumption in mango juice at elevated temperatures compared to refrigeration. The slower sugar reduction at lower temperatures corresponds with suppressed metabolic activity, supporting extended shelf life and minimal compositional changes in non‐inoculated controls.

### 3.8 Antioxidant Activity of Probiotic Jamun Juice

The antioxidant activity of the probiotic jamun juice was evaluated at Day 0 and Day 21 to assess the impact of fermentation and storage. Day 21 was selected as it corresponds to the stage at which microbial activity had stabilized, representing the final antioxidant profile shaped by microbial metabolism and phytochemical transformation. The results of the antioxidant analysis, as determined by the DPPH assay, are presented in Table 4.

**TABLE 4:** Percentage scavenging activity of probiotic and control samples at different storage temperatures (37°C, 25°C, and 4°C) on Day 0 and Day 21.

Storage duration (days)	% Scavenging activity at 37°C (incubation)		% Scavenging activity at 25°C (room temperature)		% Scavenging activity at 4°C (refrigeration)	
	Probiotic	Control	Probiotic	Control	Probiotic	Control
0	16.90 ± 0.35	16.78 ± 0.28	16.90 ± 0.35	16.78 ± 0.28	16.90 ± 0.35	16.78 ± 0.28
21	82.35 ± 1.12	68.15 ± 0.97	75.36 ± 1.08	57.08 ± 0.85	70.53 ± 0.58	43.43 ± 0.22

As shown in Table 4, probiotic samples exhibited significantly higher scavenging activity compared to their respective control samples across all storage temperatures by Day 21. The probiotic samples displayed the highest antioxidant activity at incubation temperature (37°C), followed by room temperature (25°C) and refrigeration (4°C), suggesting that elevated temperatures enhance probiotic metabolic activity, thereby improving antioxidant potential. This enhanced metabolic activity likely increases the production of antioxidant compounds such as exopolysaccharides (Sharma et al. 2024), bioactive peptides (Pereira and Gibson 2002), and enzymes (Lourens‐Hattingh and Viljoen 2001), all of which have been shown to contribute significantly to the antioxidant capacity of probiotic‐fermented products.

These results are consistent with previous studies, which report enhanced antioxidant activity in probiotics under higher temperature conditions due to increased microbial metabolism (Mishra et al. 2015).

While the control samples did show some increase in scavenging activity, their values remained consistently lower than those of the probiotics. Scavenging activity was significantly higher in treated samples compared to control samples at all temperatures on Day 21: 37°C (*p* < 0.001), 25°C (*p* < 0.001), and 4°C (*p* < 0.001), particularly under refrigeration. This emphasizes the role of probiotics in boosting antioxidant capacity. The temperature‐dependent trends observed in both probiotic and control samples further suggest that environmental conditions play a significant role in influencing antioxidant potential. Similar patterns have been reported in other studies, where higher temperatures supported greater antioxidant activity in probiotics (Østlie et al. 2005), while refrigeration resulted in reduced bioactivity (Silalahi et al. 2018).

These findings underscore the temperature‐sensitive nature of antioxidant activity in probiotic products and highlight the significant contribution of probiotics to enhancing the antioxidant potential of Jamun juice, particularly at warmer temperatures that favor microbial growth and metabolic activity.

### 3.9 Organoleptic Evaluation of Probiotic and Control Jamun Juice Samples Stored at Different Temperatures and Durations

The organoleptic evaluation of the probiotic jamun juice samples, as described in the methodology section, involved a group of 15 untrained undergraduate students aged 20–25 years using a random single‐blind method. The panelists rated each sample as either “acceptable” or “not acceptable” based on taste. The results, presented in Table 5.

**TABLE 5:** Organoleptic evaluation of probiotic and control jamun juice stored at different temperatures for 21 days.

Sample type	Storage duration (days)	Number of acceptable ratings	Number of not acceptable ratings	Percentage of acceptable ratings (%)
Control (baseline)	0	14	1	93.3 ± 6.2
Probiotic (baseline)	0	12	3	80.0 ± 10.3
Control at 37°C	21	10	5	66.7 ± 12.1
Probiotic at 37°C	21	8	7	53.3 ± 12.9
Control at 25°C	21	12	3	80.0 ± 12.1
Probiotic at 25°C	21	10	5	66.7 ± 0.9
Control at 4°C	21	12	3	80.0 ± 10.3
Probiotic at 4°C	21	11	4	73.3 ± 11.2

The organoleptic evaluation of both probiotic and control jamun juice samples stored at different temperatures for 21 days (Table 5) reveals that storage temperature conditions significantly impacted acceptability, with control samples generally receiving higher ratings than their probiotic counterparts. The probiotic samples showed a noticeable increase in acceptability at lower storage temperatures, rising from 53.3% at 37°C to 73.3% at 4°C, while control samples maintained higher sensory qualities across all storage temperature conditions, with acceptable ratings ranging from 66.7% to 80%. Notably, both sample types exhibited better preservation of acceptability when stored at lower temperatures (4°C), indicating that refrigeration may help maintain the sensory appeal of the juices over time.

However, statistical comparisons using Fisher's Exact Test (*n* = 15) showed that the differences in acceptability between control and probiotic samples were not statistically significant under any of the tested conditions (*p*‐values ranging from 0.5977 to 1.0000). The highest similarity was observed at 4°C (*p* = 1.0000), while the largest numerical gap occurred at baseline (93.3% vs. 80.0%), though still not statistically significant (*p* = 0.5977). These results indicate that although control samples tended to be more acceptable than probiotic ones, especially at higher temperatures, these differences were not strong enough to reach statistical significance at the sample size used.

Interestingly, the reduced sensory acceptability of probiotic juice at 37°C coincides with the higher antioxidant activity observed under the same condition (as discussed earlier), suggesting a potential trade‐off between functional enhancement and palatability. From a product development perspective, this highlights the importance of optimizing fermentation and storage conditions to balance health‐promoting properties with sensory quality, as consumer satisfaction remains critical for market success. Future research could explore formulation strategies, such as post‐fermentation blending or flavor enhancement, to mitigate this trade‐off.

These trends are consistent with previous research, where probiotic beverages often experience a decrease in sensory quality, such as taste, due to the presence of probiotics. For example, a study by Bayarri et al. (2010) observed that the addition of probiotics can affect taste and overall acceptability, particularly under higher temperature conditions. The present study, however, suggests that with proper temperature management, the sensory quality of probiotic beverages can be better preserved, thereby improving consumer acceptance without compromising the health benefits of probiotics.

## 4 Conclusion

This study successfully isolated *Streptococcus thermophilus* from commercial ASAS yogurt and incorporated it into jamun juice to produce a probiotic beverage. The growth and metabolic activity of *S. thermophilus* were temperature‐dependent, with rapid fermentation observed at 37°C, resulting in significant reductions in sugar content and enhanced antioxidant activity. Lower storage temperatures (25°C and 4°C) helped preserve the probiotic's viability and extended shelf life, with minimal impact on sensory quality. Probiotic jamun juice exhibited increased antioxidant potential, particularly under warmer storage temperature conditions, demonstrating the beneficial role of probiotics in functional beverages. This study highlights the importance of optimizing storage temperature conditions to maintain both the health benefits and sensory appeal of probiotic drinks. Future studies could focus on exploring the long‐term stability and sensory characteristics of probiotic jamun juice under varied storage temperature conditions, as well as conducting a comprehensive analysis of other nutrients, such as vitamins, minerals, proteins, and bioactive compounds, to further elucidate the nutritional and health benefits of probiotic functionalized beverages.

## Author Contributions

**Dorcus Nnko:** conceptualization (supporting), data curation (lead), formal analysis (equal), funding acquisition (lead), investigation (equal), methodology (equal), resources (supporting), validation (equal), visualization (equal), writing – original draft (lead), writing – review and editing (supporting). **Ally Mahadhy:** conceptualization (lead), data curation (supporting), formal analysis (equal), funding acquisition (supporting), investigation (supporting), methodology (equal), project administration (lead), resources (lead), supervision (lead), validation (equal), visualization (equal), writing – original draft (supporting), writing – review and editing (lead).

## Ethics Statement

This study did not involve the use of live animals. The probiotic bacteria used to functionalize the probiotic jamun juice were isolated from commercially available ASAS yogurt purchased from a local market in Dar es Salaam. The study received ethical approval from the University of Dar es Salaam (UDSM) through the Department of Molecular Biology and Biotechnology and the University's Ethical Committee. Sensory evaluation was carried out with the voluntary participation of 15 undergraduate students from UDSM, in accordance with ethical guidelines and after obtaining informed consent.

## Consent

All participants involved in the sensory evaluation provided informed consent prior to their participation. The process adhered to the ethical guidelines approved by the University of Dar es Salaam.

## Conflicts of Interest

The authors declare no conflicts of interest.

## Data Availability

Raw data will be available on request.
